# Understanding ageing: Biomedical and bioengineering approaches, the immunologic view

**DOI:** 10.1186/1742-4933-5-9

**Published:** 2008-09-03

**Authors:** Matteo Bulati, Mariavaleria Pellicanò, Sonya Vasto, Giuseppina Colonna-Romano

**Affiliations:** 1 Gruppo di Studio sull'immunosenescenza and Dipartimento di Biopatologia e Metodologie Biomediche, Università di Palermo, Palermo, Italy; 2 Unità Operativa di Immunoematologia e Medicina trasfusionale, Università di Palermo, Palermo, Italy

## Abstract

During the past century, humans have gained more years of average life expectancy than in the last 10,000 years; we are now living in a rapidly ageing world. The sharp rise in life expectancy, coupled to a steady decline in birth rates in all developed countries, has led to an unprecedented demographic revolution characterized by an explosive growth in the number and proportion of older people. Ageing is a complex process that negatively impacts the development of the immune system and its ability to function. Progressive changes in the T and B cell systems over the life span have a major impact on the capacity to respond to immune challenge. These cumulative age-associated changes in immune competence are termed Immunosenescence: some immunological parameters are commonly notably reduced in the elderly and, reciprocally good function is tightly correlated to health status. Hence, a better understanding of Immunosenescence and the development of new strategies to counteract it are essential for improving the quality of life of the elderly population.

## Background

The "Understanding Aging: Biomedical and Bioengineering Approaches" conference was held from June 27–29, 2008 at UCLA organized by Aubrey de Grey to discuss and talk about possible intervention in ageing.

During the past century, mankind has gained more years of average life expectancy than in the last 10,000 years. We are now living in a rapidly ageing world. The sharp rise in life expectancy, coupled to a steady decline in birth rates in all developed countries, has led to an unprecedented demographic revolution characterized by an explosive growth in the numbers and proportion of older persons. Nowadays, people are living much longer than they used to and the longer they live, the longer their bodies are exposed to environmental factors which increase the risk of age-associated diseases. The reduction of the response to environmental stimuli is associated with an increased predisposition to illness and death. This progression causes a reduction of the response to environmental stimuli and, in general, is associated with an increased predisposition to illness and death. In Western countries, the mortality rate increases in people over 65 years, if compared with individuals between 25 and 44 years old, by 100-fold for stroke, as well as chronic lung disease, 92-fold for heart disease, 89-fold for pneumonia and influenza, 43-fold for cancer [1]. On the contrary, ageing in good condition seems directly correlated with a good functioning of the immune system, suggesting that there are genetic determinants of longevity in genes regulating the immune inflammatory response [2,3].

In senescence alterations of innate and instructive immunity have been described. The modifications of the immune system in the elderly are generally evaluated as a deterioration of the immune system, this is the origin of the term immunosenescence. A good immune system in the elderly is tightly correlated to health status, and some immunological parameters are often notably reduced in the elderly. On the other hand infectious diseases, tumors, autoimmune phenomenona and inflammatory chronic diseases like atherosclerosis and Alzheimer's disease, are frequent in this phase of the life course [3-5].

A body of experimental and clinical evidence has suggested that the immune system is implicated, with a variable degree of importance, in almost all age related or associated diseases. Both innate and instructive immune systems are usually involved in the pathogenesis of these chronic diseases. However, innate immunity appears to be the prevalent mechanism driving tissue damages associated with different age-related diseases [6]. So, ageing is accompanied by an age-dependent up-regulation of the inflammatory response, due to the chronic antigenic stress that impinges throughout life upon innate immunity, and has potential implications for the onset of inflammatory diseases [7].

Here is an extract of the talks and posters presented:

### Mitochondrial damage

Perturbation of mitochondrial Fe homeostasis cause a decline in mitochondrial function that causes neuromuscular degenerative disease and other tissue dysfunction. C. Leeuwenburgh suggested that mitochondrial non-heme Fe represents a potential novel target for targeted interventions to slow ageing (C. Leeuwenburgh, University of Florida, USA) [8].

### Micronutrient inadequacy

It was proposed that inadequate micronutrient intake leads to metabolic modification that has long term consequences such as cancer (DNA damage), severe infection (immune dysfunction) and cognitive dysfunction and accelerated ageing (mitochondrial decay). Much evidence supports the idea that micronutrient shortage accelerate ageing (B.N. Ames, University of California, Berkeley, CA, USA) [9].

### Telomeres

The shortening of telomeres is supposed to be the molecular clock of ageing; indeed there is a strong correlation between age and telomere length and shorter telomeres directly correspond to shorter human life expectancy. For this purpose, several biotech organizations have accepted the challenge of finding ways to prevent telomere shortening by transiently inducing the activity of telomerase (L.A. Briggs, Reno, NV, USA). Another research has shown that therapy acting on the catalytic component of human telomerase, such as TAT2, a small molecule telomerase activator, could stabilize the telomere length and retard the loss of the immune control over viral infection (R. Effros, UCLA, Los Angeles, CA, USA) [10].

### Immunological Point of view

On the immunological side Dr Z. Cui has shown that cancer cells in vitro could be killed by the effector cells of the innate immune system such as macrophages and neutrophils. A similar activity was discovered in some healthy people concerning granulocytes and monocytes (Z. Cui, Winston-Salem, NC, USA) [11].

According to the fact that the immune system plays an important role in ageing, the group of Prof. C. Caruso, actively involved in immunosenescence studies [2-5], has demonstrated that B naïve lymphocytes, are increased in the offspring of healthy old centenarians. It has been demonstrated that the children of centenarians, who are in their 70s and 80s, have a survival advantage when compared with control subject of the same age range whose parents died at an average life expectancy [3]. The main lymphocyte differences observed between the two groups concern B cells. Indeed naïve B cells are more abundant as well as double negative B cells in centenarian children. These data are similar to that found in previously experiment on young subjects. So, B cell compartment of the offspring of centenarians seems to be more similar to that of young respect to the old one (S. Vasto, University of Palermo, Italy) [12].

It is well known that change in immune function are hallmarks of ageing and the group of Dr. A. Agrawal (University of California, Irvine, CA, USA) has shown that the reactivity of dendritic cells to self-antigens can be characteristic of ageing features. Furthermore, this over-reactivity induces lymphocyte T proliferation with subsequent higher risk of autoimmune diseases [13].

Interestingly, Effros's group suggests a possible involvement of hyper-activated T cells in bone loss associated with vascular disease in aged mice. The increased proportion of CD8 T cells lacking expression of the co-stimulatory receptor CD28 leads to decreased vaccine responsiveness and early mortality [14]. ST Parish found that loss of CD28 expression is caused by increased Caspase-3 activity that can be induced by Tumor necrosis factor-alpha and suggested possible strategies for retarding the generation of senescent CD8 T cells during ageing (L.S. Graham, UCLA, Los Angeles, CA, USA).

## Conclusion

Ageing is a complex process that negatively impacts the development of the immune system and its ability to function. Progressive changes in the T and B cell systems over the life span have a major impact on the capacity to respond to immune challenge. These cumulative age-associated changes in immune competence are termed immunosenescence. A better understanding of immunosenescence and the development of new strategies to counteract it are essential for improving the quality of life of the elderly population [5]

## Competing interests

The authors declare that they have no competing interests.

## Authors' contributions

All authors contributed equally to the paper and read and approved the final manuscript
